# Contrasting MEG effects of anodal and cathodal high-definition TDCS on sensorimotor activity during voluntary finger movements

**DOI:** 10.3389/fnimg.2024.1341732

**Published:** 2024-02-05

**Authors:** Jed A. Meltzer, Gayatri Sivaratnam, Tiffany Deschamps, Maryam Zadeh, Catherine Li, Faranak Farzan, Alex Francois-Nienaber

**Affiliations:** ^1^Rotman Research Institute, Baycrest Academy for Research and Education, Toronto, ON, Canada; ^2^Departments of Psychology and Speech-language Pathology, University of Toronto, Toronto, ON, Canada; ^3^School of Mechatronic Systems Engineering, Simon Fraser University, Burnaby, BC, Canada

**Keywords:** magnetoencephalography (MEG), TDCS, MCRF, polarity, beta oscillations, gamma oscillations, motor cortex

## Abstract

**Introduction:**

Protocols for noninvasive brain stimulation (NIBS) are generally categorized as “excitatory” or “inhibitory” based on their ability to produce short-term modulation of motor-evoked potentials (MEPs) in peripheral muscles, when applied to motor cortex. Anodal and cathodal stimulation are widely considered excitatory and inhibitory, respectively, on this basis. However, it is poorly understood whether such polarity-dependent changes apply for neural signals generated during task performance, at rest, or in response to sensory stimulation.

**Methods:**

To characterize such changes, we measured spontaneous and movement-related neural activity with magnetoencephalography (MEG) before and after high-definition transcranial direct-current stimulation (HD-TDCS) of the left motor cortex (M1), while participants performed simple finger movements with the left and right hands.

**Results:**

Anodal HD-TDCS (excitatory) decreased the movement-related cortical fields (MRCF) localized to left M1 during contralateral right finger movements while cathodal HD-TDCS (inhibitory), increased them. In contrast, oscillatory signatures of voluntary motor output were not differentially affected by the two stimulation protocols, and tended to decrease in magnitude over the course of the experiment regardless. Spontaneous resting state oscillations were not affected either.

**Discussion:**

MRCFs are thought to reflect reafferent proprioceptive input to motor cortex following movements. Thus, these results suggest that processing of incoming sensory information may be affected by TDCS in a polarity-dependent manner that is opposite that seen for MEPs—increases in cortical excitability as defined by MEPs may correspond to reduced responses to afferent input, and vice-versa.

## Introduction

Application of low intensity electrical currents directly to the scalp, termed transcranial direct-current stimulation (TDCS), can induce changes in cortical excitability that may last for over an hour after stimulation (Caparelli-Daquer et al., 2012; Kuo et al., 2013). Changes in “cortical excitability” are operationally defined as changes in the size of motor-evoked potentials (MEPs) in peripheral muscles, elicited by single pulses of transcranial magnetic stimulation (TMS) applied to the motor cortex. Modulation of MEPs has proven to be a useful basis for characterizing noninvasive brain stimulation (NIBS) protocols as excitatory (increasing MEPs) or inhibitory (decreasing MEPs). However, this measure is completely dependent on the peripheral output of the motor cortex, and thus it is difficult to generalize it to other parts of the cortex, which lack a direct “output signal” by which to quantify excitability. In most MEP-based studies of TDCS, anodal stimulation (positive electrode positioned over the motor cortex) is excitatory, while cathodal stimulation (negative electrode) is inhibitory (Jacobson et al., 2012).

Despite the limitations, the characterization of protocols as excitatory and inhibitory on the basis of MEPs has been widely influential, and brain stimulation protocols are widely assumed to have similar effects on other regions of the cortex when they are applied in research studies. The accuracy of this generalization is a critical assumption behind a large and ever-growing body of research. Unfortunately, studies of the physiological and cognitive effects of TDCS have failed to converge on a conclusion consistent with the idea that it can be used to “turn up” and “turn down” the engagement of a given brain area as desired (Jacobson et al., 2012; Horvath et al., 2015). Furthermore, NIBS is increasingly being used in the treatment of disorders such as stroke and depression, with putatively excitatory and inhibitory protocols being applied to opposite hemispheres to correct suspected imbalances in transcallosal inhibition between homologous regions (Chrysikou and Hamilton, 2011; Blumberger et al., 2016). As research and clinical applications of the technique expand, it is more important than ever to have a means of evaluating the physiological effects of NIBS protocols beyond the motor cortex.

Because the changes in membrane excitability evoked by TDCS, as assessed by MEPs, may in fact be a product of the specific circuitry of the motor cortex, it is desirable to develop physiological assessments of the after-effects of TDCS that can be applied to any arbitrary region. Of course, for any such measure, the logical starting point would be to apply it to the motor cortex, where the effects on MEPs are already well characterized. Thus far, the most popular techniques for physiological measurement of TDCS effects have been functional magnetic resonance imaging (fMRI) and electroencephalography (EEG). A major limitation of fMRI is that it does not have an interpretable absolute signal, and is instead based on comparing relative signal levels between two or more states. It is uncertain what effect an increase in cortical excitability may have on task-induced activation of the motor cortex or any other area. In general, one can distinguish two possible scenarios. First, increasing cortical excitability, as measured by MEPs, may also correspond to an increase in task-induced activation given the same task demands, leading to a positive relationship between the two measures. In this scenario, cortical excitability may correspond to an overall “gain” factor; we therefore refer to it as the “neural gain” hypothesis. In contrast, another plausible scenario is that increased cortical excitability would correspond with reduced task-induced activation. For example, the metabolic and hemodynamic demand of the area may be raised at baseline due to excitatory stimulation, without a change in the demand induced by the task, such that the observed increment from task-induced activation may be smaller, leading to a negative relationship between MEPs and task-induced activation. In keeping with findings that reduced task-induced signals are linked to greater efficiency and improved performance following training (Gobel et al., 2011; Deery et al., 2023) we refer to this as the “neural economy” hypothesis.

Studies combining fMRI with TDCS have yielded results consistent with both hypotheses. Studies supporting the neural gain hypothesis include: Baudewig et al. (2001) and Stagg et al. (2009), while Meinzer et al. (2013) supported the neural economy hypothesis. Some studies have failed to find consistent effects of TDCS on motor cortex activation (Kwon et al., 2008; Antal et al., 2011).

Compared to fMRI, EEG may offer a more nuanced picture of dynamic neural activity, as multiple complementary measures can be derived from it. These include, among others: (1) spontaneous oscillations, (2) event-related potentials (ERPs), or time-domain average voltage changes that are time-locked to specific events, and (3) event-related spectral perturbations (ERSPs), i.e., increases and decreases in the power of oscillations induced by a specific event. A disadvantage of EEG, however, is the relatively poor spatial resolution, which makes it difficult to localize observed signals to specific brain regions such as the motor cortex. A promising alternative is magnetoencephalography (MEG), which offers access to the same rich dynamics as EEG, but allows for more accurate reconstruction of signals in source space (Hamalainen, 1993). This improved spatial resolution is due to the transparency of the skull to magnetic fields, giving greater accuracy to the calculations that estimate the projection of neural currents to external sensors (the “forward solution”) and subsequently the estimation of those intracranial currents on the basis of external measurements (the “inverse problem”).

MEG studies of voluntary finger movements have demonstrated a set of distinct signals that are reliably induced by individual movements and specifically localized to motor cortex. In the time-frequency domain, these include an event-related desynchronization (ERD, or power decrease) in the beta frequency band (~15–35 Hz), which is bilateral but stronger in contralateral compared to ipsilateral cortex, a later “beta rebound” event-related synchronization (ERS, i.e., power increase above baseline) also predominant in contralateral motor cortex, and a brief ERS in the gamma range (65–85 Hz) occurring immediately after movement onset and also predominantly contralateral (Cheyne, 2013). Examples of these signals can be seen in **Figure 2** of this paper. In the time-domain, MEG studies have revealed multiple peaks in the average signal time-locked to movement onset, termed Event-related fields (ERF), all of which are localized to sensorimotor cortex contralateral to the moving hand. These ERFs, sometimes termed movement-related cortical fields (MRCF), sometimes include components seen before the movement, especially for self-paced voluntary movements, including a slow “readiness field” detectable up to several seconds before the movement, and a “motor field,” a faster peak seen immediately before the movement onset (Kristeva et al., 1991). Other MCRF components, reliably detected after movement onset whether self-paced, cued, or even passive, are called “motor-evoked fields” (MEF) and are thought to result from proprioceptive feedback conveyed to motor cortex involved in motor control. Studies examining MEFs have revealed up to three distinct peaks, termed MEFI, MEFII, and MEFIII, which have different magnitudes, and may have different polarities reflecting distinct sources of input to cortex. These three peaks tend to colocalize to the same region within the central sulcus, although studies have differed in ascribing these components to post-central sensory areas or pre-central motor areas (Kristeva-Feige et al., 1996; Cheyne et al., 1997, 2006; Woldag et al., 2003; Murakami et al., 2010; Onishi et al., 2013; Suzuki et al., 2013). Examples of MRCFs can be seen in the time-frequency domain in **Figure 2**, and in the time domain in **Figure 3**.

All of these signals, in addition to spontaneous oscillations recorded at rest, might in principle be modulated by brain stimulation. Furthermore, similar signals to these are present in other cortical regions beyond the motor cortex when those areas are activated by relevant task demands (Neuper and Pfurtscheller, 2001; Hanslmayr et al., 2016). Thus, characterization of the effects of putatively excitatory and inhibitory NIBS protocols on these task-induced signals within the motor cortex has the potential to generalize to the rest of the cortex. This allows us to empirically test the assumption that the task-related engagement of a given region can be modulated positively and negatively by appropriate selection of stimulation protocols.

To date, only very limited attempts have been made to characterize the after-effects of TDCS on voluntary motor activity using MEG. Soekadar et al. (2013) demonstrated the feasibility of measuring motor activity with beamforming during simultaneous application of TDCS in the MEG scanner, and Hanley et al. (2016) demonstrated enhanced ERFs and reduced gamma ERS *during* (not following) 10 min of TDCS at 1 mA intensity. Similarly, Garcia-Cossio et al. (2016) demonstrated increased readiness fields preceding movements *during* anodal TDCS. However, few studies have yet characterized the after-effects on motor cortex dynamics of the most common form of TDCS used to modulate cortical excitability in both motor and cognitive studies—i.e., 20 min of stimulation followed by performance of the experimental task, despite the fact that it is the after-effects (20–30 min following stimulation) that have led to the widespread characterization of different stimulation protocols as excitatory or inhibitory.

In the present study, we aimed to characterize the after-effects of anodal and cathodal TDCS by examining the modulation of well-characterized time and time-frequency domain responses that occur during cued voluntary finger movements. To improve the spatial precision of our results, we used MEG to localize electrophysiological activity within the motor areas that directly control finger movements. Additionally, we used high-definition TDCS (HD-TDCS), a refinement of traditional TDCS that employs multiple small electrodes to concentrate electrical current on a specific brain area. An HD-TDCS montage typically involves a 4 × 1 center-surround array, in which the central electrode is placed on the target area and four electrodes of opposite polarity to the central one divide the return current. HD-TDCS has been shown to have similar effects on MEPs as conventional TDCS, with a central anode being excitatory and a central cathode being inhibitory (Kuo et al., 2013). Here, participants performed voluntary visually cued movements of the left and right index finger, before and after HD-TDCS applied to the left primary motor cortex. We focused our analysis on the detection of polarity-dependent contrasting effects of anodal TDCS (aTDCS) and cathodal TDCS (cTDCS), in order to determine whether the neural gain or neural economy hypothesis applies to electrophysiological activity associated with voluntary movements. Under the neural gain hypothesis, anodal stimulation should increase the magnitude of task-induced neural responses, while cathodal stimulation should decrease it, and under the neural economy hypothesis, the reverse should be true. Given that the existing literature contains support for both hypotheses across studies (see discussion for examples), we did not have a strong expectation about the directionality of the outcome for the present study.

## Methods

### Participants

Nineteen participants (six male, 13 female, mean age: 24.3 years, SD 5.2) were recruited from the University of Toronto and Baycrest Hospital communities. All self-reported as strongly right-handed, and had no history of neurological disorders, syncope, or chronic migraine (Rossi et al., 2009). All participants were screened for the presence of metal on the body. Furthermore, we tested the quality of the MEG signal of subjects who reported any kind of dental work (except tooth fillings) prior to their first session, to ensure that the MEG signals were minimally contaminated by artifacts attributable to metal in the mouth. One participant had poor-quality EMG data precluding accurate identification of finger movement onset, and was thus excluded from the analysis of movement-related activity, but was included in the analysis of resting state data. Each participant completed three experimental sessions. We collected anatomical MRI data during the first session, which typically lasted a half hour. The participants then came back for two TDCS-MEG sessions, that were counter-balanced so that half received cTDCS in the first session and aTDCS in the second session, while the order was reversed for the others. All sessions were at least 48 h apart (mean days apart: 16.4, SD 23). Some of the data collected in this study was used for a previous publication investigating methods for interhemispheric connectivity in MEG (Wei et al., 2021). The data in that paper was taken only from the pre-stimulation timepoint, and also included additional participants who participated in a similar study (not yet published) involving a different neurostimulation technique.

### MRI acquisition

A high-resolution (192 × 256 × 160 voxels; 1 mm^3^ isotropic voxels) T1-weighted MP-RAGE structural scan was collected for each subject on a 3 T MRI system (Siemens Magnetom Trio) located at Baycrest.

### MEG finger movement task

**Figure 1:**
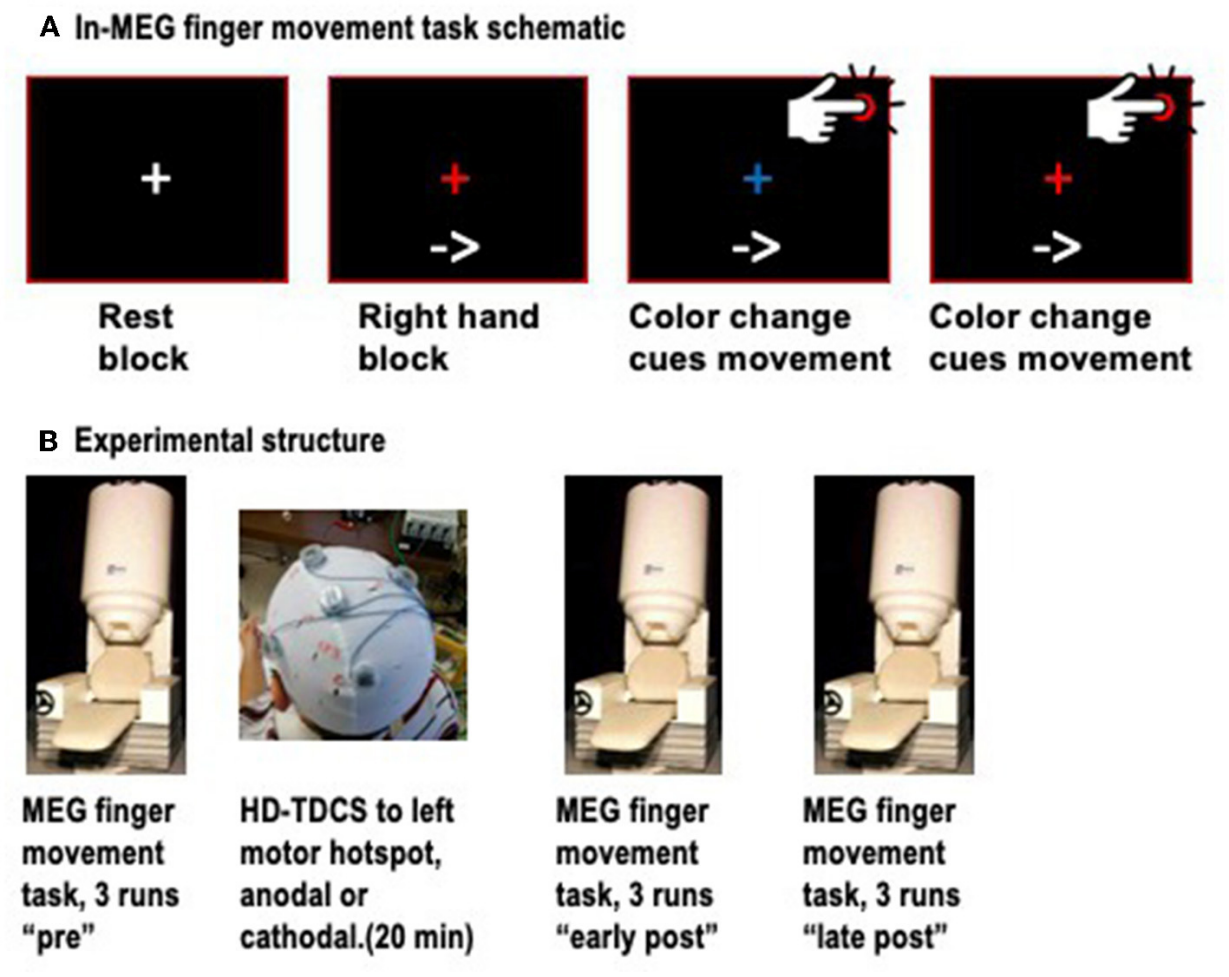
**(A)** Schematic of the finger movement task, showing a resting block (white fixation cross), and finger movement blocks, in which an arrow at the bottom continuously indicates which hand to use, and color changes of the fixation cross between red and blue cue the individual finger movements. **(B)** Schematic of the timepoints, with three runs of MEG conducted before HD-TDCS, and six runs after, divided into early and late post-TDCS timepoints.

Neuromagnetic signals were recorded with a 151-channel whole-head MEG system (CTF MEG, Coquitlam, BC, Canada). Detection coils for this system are configured as first-order axial gradiometers in hardware, and as synthetic third-order gradiometers in software to reduce external noise (Vrba and Robinson, 2001). Continuous MEG data were acquired at 625 Hz and low-pass filtered at 200 Hz for each run, concurrently with EMG activity in the first dorsal interosseous (FDI) muscles of both hands, for accurate detection of the onset of finger movements. Head position in the helmet was tracked with three fiducial coils and measured at the start and end of each run. To minimize head movements, we used a small towel or pillow case to provide a snug fit within the MEG helmet while keeping the cortex as close to the sensors as possible. Participants were seated on a padded chair, with both arms resting on armrests and the index fingers resting on top of a push-button. The task was divided into “runs” of duration 6 min, 40 s, each consisting of four rest blocks, four blocks of left finger movements, and four blocks of right finger movements, arranged in a pseudo-random order. Rest blocks were 10 s long, while task blocks were ~40 s long. At the start of each block, participants viewed a visual text cue, saying “Rest,” “Left hand,” or “Right hand.” In rest blocks, participants simply viewed a white central fixation cross. For finger movement blocks, an arrow pointing to the left or right also appeared, and remained throughout the block to ensure that participants would not forget which hand to use during the block. In finger movement blocks, participants viewed a central fixation cross that alternated color between red and blue at ~4-s intervals, with a random jitter of ± 500 ms to prevent participants from predicting the exact onset of the color change. Participants were instructed to press the button with the index finger of the active hand immediately upon detecting the color change (Figure 1A). Three runs of this task were completed (~21 min of recording) prior to the administration of HD-TDCS. Next, participants left the MEG and proceeded to the adjacent stimulation room, where they received HD-TDCS. Immediately following the HD-TDCS, participants returned to the MEG and completed six more task runs. The mean transfer time from HD-TDCS back to MEG was 5:56 ± 2:18 min. Given that the effects of TDCS on motor cortex excitability are known to last about 30–60 min after stimulation, we divided these runs into “early post-” and “late post-” periods for analysis purposes, covering ~5–30 min and 30–55 min after the end of the stimulation (Figure 1B).

### Motor hotspot localization

To identify the brain area controlling index finger abduction, the intended target of HD-TDCS, we applied single-pulse TMS to find the motor “hotspot,” the site eliciting maximum motor-evoked potentials (MEPs) in response to pulses. TMS pulses were delivered with a 70 mm figure-eight coil attached to a biphasic stimulator (Magstim Super Rapid^2^ Plus). A neuronavigation system (Brainsight, Rogue Research) was used to monitor coil position relative to the subject's brain, using a 3D head model derived from their individual structural MRI scan.

TMS pulses to the left motor cortex were delivered while recording electromyogram (EMG) activity from the FDI muscle of the right hand. The TMS coil was held at approximately a 45-degree angle relative to the midline, with coil facing anteriorly. Different locations within the left precentral gyrus were probed to find the maximally responsive spot. Once found, the spot was marked with an erasable skin marker. We chose the FDI muscle for this experiment because it is easy to record a clear EMG signal from it, both when localizing the hand motor cortex with TMS, and when detecting the onset of button press movements during MEG. Even though the primary function of the FDI muscle is for lateral finger and thumb movements, it also participates in the vertical movements involved in button pressing with the index finger and is easy to detect. In contrast, the flexor digitorum superficialis muscle, which is primarily responsible for vertical movements, is much larger and located deeper within the forearm, making it a less reliable source of surface EMG measurements to detect and quantify finger movements.

### HD-TDCS administration

HD-TDCS was applied to motor cortex using 2 mA of current in a standard 4 × 1 montage (Bikson et al., 2016; Woods et al., 2016; Antal et al., 2017), with four surround electrodes carrying 0.5 mA and one central return electrode carrying 2 mA, according to a published protocol (Villamar et al., 2013). Electrode holders (HD1, Soterix Medical Inc.) were placed into an elastic cap (Easycap Gmbh) worn by the participant, with the position adjusted such that the central electrode lay directly over the marked motor hotspot. Conductive gel (Signagel) was inserted into each electrode holder, and Ag/AgCl ring electrodes were placed inside (Minhas et al., 2010). A cotton swab was used to move aside hair to ensure acceptable impedances (<5 k, measured with a portable impedance meter). Next, the electrodes were attached to a multichannel transcranial electrical stimulator (DC-Stimulator MC, Neuroconn). For aTDCS, the central electrode was the anode, with four cathodal return electrodes, and for cTDCS, the opposite. Stimulation was delivered at the target level for 20 min, plus a 30-s period of current ramp-up at the beginning and 30 s of ramp-down at the end. To provide a fairly consistent level of cognitive and sensory activity during the stimulation, all participants viewed one episode of a television program during each stimulation session (“The Simpsons,” selected from season 20, aired in 2009). No participants reported having previously seen the episodes selected for the experiment. All participants filled out a questionnaire about adverse effects after the experiment; none were noted other than mild skin tingling and itching at the stimulation site, consistent with other investigations (e.g., El Jamal et al., 2023).

### EMG processing

To identify the temporal onset of finger movements, we analyzed the EMG of both FDI muscles. The EMG was high-pass filtered at 20 Hz, rectified, and then low-pass filtered at 30 Hz (Abbink and Glas, 1998). The onset of EMG activity was semi-automatically identified in each trial by a human rater supported by a Matlab script that suggested an onset automatically using a threshold-based algorithm (Lidierth, 1986; Van Boxtel et al., 1993). The human rater could override the script to adjust the onset when necessary, and remove epochs that seemed very different from the rest—for example, we removed trials in which there were two large and distinct EMG components, as it wasn't clear if a single and continuous finger movement had been performed. Thus, we only analyzed trials in which an unambiguous EMG onset following the visual cue could be identified (86% of all trials). Statistical analysis of reaction time was conducted in R 4.2.2, using the “ez” package for repeated measures ANOVA and the emmeans package for *post-hoc* pairwise contrasts. We conducted a repeated measures ANOVA including within-subject factors of hand, stimulation type, and timepoint (see results).

### MEG preprocessing

To construct head models for MEG analysis, the locations of the fiducial points were marked manually in AFNI software, and the T1-weighted MRI was spatially transformed into the coordinate space of the MEG data. The skull was stripped using 3DSkullStrip in AFNI, and a 3-D convex hull approximating the inner surface of the skull was constructed using the NIH software package Brainhull. Taking into account the position of the head relative to the sensors, a multi-sphere model (Huang et al., 1999) was computed. To normalize MEG source estimates into MNI space, we computed a nonlinear warp of each subject's brain to the MNI152 standard-space T1-weighted average structural template image using the software package ANTS (Avants et al., 2011). This warp was then used to transform single-subject MEG activity maps into MNI space for multi-subject statistical analysis.

All analyses of MEG data other than initial artifact screening were conducted in source space, estimating the time course of activity at specific intracranial locations using the beamforming algorithm Synthetic Aperture Magnetometry (SAM). For each desired location, SAM constructs an optimized spatial filter that estimates a virtual signal of electromagnetic activity generated at the target location while attenuating activity arising from anywhere else (Van Veen et al., 1997; Vrba and Robinson, 2001). The spatial filter is constructed from the data covariance matrix and a lead field map derived from the MRI head model. While SAM is overall similar to other beamformers, it includes a nonlinear optimization step to fix the orientation of the reconstructed dipole at each location to maximize sensitivity. Analysis of MEG signals in source space has multiple advantages over direct analysis of sensor data. First, beamformed source space signals are relatively insensitive to artifacts generated by muscles outside the brain, with the possible exception of ocular signals leaking into the orbito-frontal cortex (Bardouille et al., 2006). Second, analysis of source-localized virtual channels compensates for individual differences in head position and brain shape with respect to the MEG sensors, whose location is fixed in the helmet. Third, beamforming produces an estimate of neural currents in the brain, whereas sensor signals depend strongly on the design of the sensor and vary greatly in character across different types of MEG machine.

MEG data were first screened for artifacts manually. We removed trials with large, visually obvious disruptions in the signal such as coughs or a sudden large head movement (<1% of all trials). We did not exclude trials on the basis of minor artifacts such as eye blinks, saccades and muscle tension, as these are easily projected out of the reconstructed cortical activity time courses in the beamforming process (Vrba, 2002). Head position during a run was determined as the average of the positions recorded at the start and end of the run, and the head position was then averaged across three runs comprising the pre-stimulation, early post-stimulation, and late post-stimulation sessions (mean RMS across sessions and subjects : 2.86 mm, SD 1.50). Adaptive beamforming methods are also robust to small head movements (Robinson and Vrba, 1999).

### Conversion to source-level signals

Beamforming analysis is commonly done in either of two ways: whole-brain imaging of differences in oscillatory power between two conditions, and reconstruction of the full time course of source-space activity in specific locations. Due to computational constraints, it is not generally feasible to do a full reconstruction of millisecond-level activity on the whole brain at once. Whole-brain contrasts therefore require pre-selection of the time and frequency windows of interest. Fortunately, the oscillatory dynamics of the motor cortex during unilateral finger movements have already been extensively characterized in the literature (Neuper and Pfurtscheller, 2001; Cheyne, 2013). Based on these previous findings, we selected the following three well-known event-related changes in power: (1) Event-related desynchronization (ERD, or power decrease) in the beta band (15–35 Hz), in the time window of 0–400 ms after EMG onset, (2) post-movement beta band event-related synchronization (ERS, or power increase), sometimes termed “beta rebound” as it exceeds baseline power levels after a movement is complete, in the time window of 550–1,250 ms, and gamma-band ERS (65–85 Hz) in the time window of 50–250 ms.

Whole-brain contrasts were computed at all voxels in a 5 mm grid using the SAM pseudo-T value (Robinson and Vrba, 1999), a normalized estimate of the change in band power relative to a time window of equivalent length in the baseline period (before the visual cue to move). For each contrast, whole-brain maps of pseudo-t values were warped into MNI space for multi-subject analysis.

To confirm that the chosen time-frequency windows were appropriate for the present data set, we also reconstructed full timecourses of activity in left and right motor cortex for subsequent time-frequency analysis. As seen in prior studies, whole-brain maps consistently revealed unilaterally dominant activity centered in the precentral gyrus for beta ERS and gamma ERS (Gaetz et al., 2011; Cheyne, 2013), with higher spatial precision for gamma (a sharper peak), but higher consistency for beta, as some subjects did not show a distinct gamma response on both sides. In contrast, beta ERD produced maps that were much more symmetrically bilateral, with a more posterior distribution covering both precentral and postcentral gyrus (Kilavik et al., 2012). Thus, for localizing virtual channels representing motor activity, we selected the precentral voxel showing maximal gamma ERS in each subject, or maximal beta ERS if the peak gamma ERS voxel did not fall within the precentral gyrus. Beamformer weights were then computed for each location on 0–100 Hz bandpass filtered sensor data from all the trials. We then multiplied single-trial sensor activity by the weights to reconstruct source current activity at both virtual channels (Robinson and Rose, 1992).

**Figure 2:**
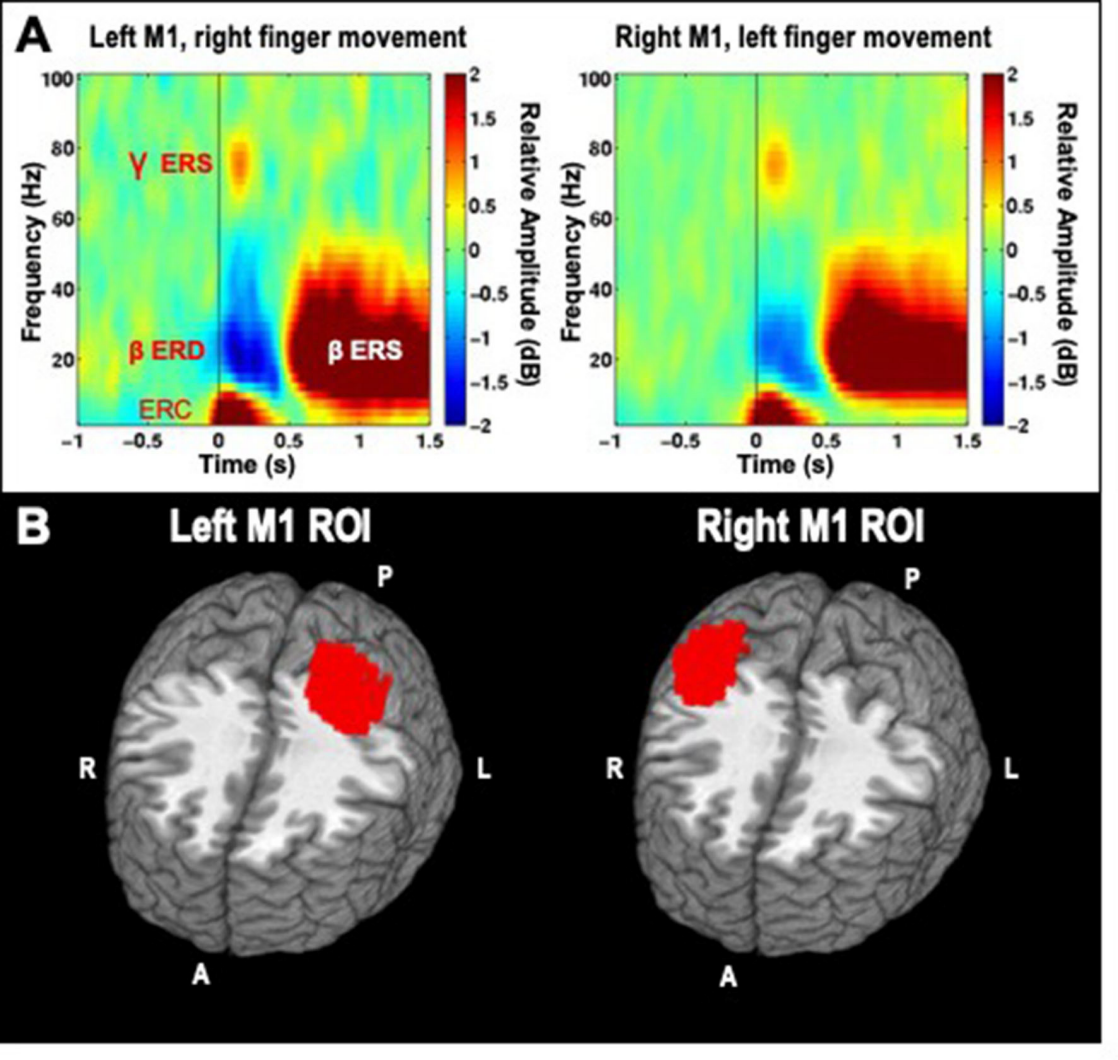
**(A)** Time-frequency decompositions of MEG virtual channel signals averaged across participants, for right and left hand finger movements, showing gamma event-related synchronization (ERS), beta event-related desynchronization (ERD), beta rebound ERS, and low-frequency event-related currents (ERC), which are further analyzed through subsequent time-domain averaging. Data is taken from the pre-stimulation timepoint of the cathodal TDCS session. **(B)** Left and Right ROIs for primary motor cortex (M1).

We analyzed induced spectrotemporal changes in the virtual channel data using a short-time discrete Fourier transform. The average log-power in the baseline period for both left and right finger movements were used as a common baseline, and subtracted from the log-power at each time-frequency point. These values are then averaged to produce event-related spectral perturbation (ERSP) spectrograms (Makeig, 1993) for each channel and condition (left or right movement). This procedure ensures that differences between conditions cannot be attributable to differences in the baseline, as the same baseline power values are used across both conditions. ERSPs were visually inspected as a quality control step, pre-stimulation group averages for contralateral movements are presented in Figure 2A, and demonstrate that the chosen time-frequency windows are a good match for the observed data.

### Regions of interest

Although the virtual channel procedure described above allows for detailed analysis of neural signal in source space, it is limited to a single location at a time, and may therefore miss or underrepresent relevant changes induced by HD-TDCS. Therefore, we selected larger regions of interest (ROIs) to summarize activity in the portion of motor cortex activated by unilateral finger movements, averaging together activity estimates derived from whole-brain analysis across all voxels within the ROI. As beta ERS was a very reliable signal to locate cortical tissue involved in finger movements, we averaged together all of the beta ERS whole-brain maps across subjects and conditions. A simple nearest-neighbors algorithm was then used to construct a 150 voxel cluster (5 mm^3^ voxels) consisting of voxels with the highest average pseudo-t values for the left M1, based on the right finger data, and another 150 voxel cluster-based ROI for the right M1, based on the left finger data (Figure 2B).

### Event-related currents

In addition to the ERD and ERS signals discussed above, finger movements also elicit an increase in power at low frequencies (0–10 Hz), in a time window of 0–0.5 s, seen in Figure 2A. This low-frequency signal is not generally a periodic oscillation, but rather an increase in power driven by specific event-related signals that are reproducibly phase-locked to the event-onset, corresponding to ERPs or ERFs. Because we are analyzing the source-space equivalent based on reconstructed currents, we refer to them as event-related currents (ERCs). The full time course of ERCs can be characterized in source space using the computationally efficient method of event-related SAM (Cheyne et al., 2006). Because they are simply the result of multiplying the sensor data averaged across trials by the beamformer weights, one can readily obtain an estimated timecourse at every voxel in the brain. After downsampling the sensor data to 125 Hz and band-passing at 0–20 Hz, we computed beamformer weights using single-trial data for voxels on a 7 mm 3D grid covering the whole brain. The spatial and temporal downsampling improved computational efficiency. Because the polarity of ERC signals can also vary arbitrarily across voxels and subjects depending on the orientation of the dipole chosen by the SAM beamformer, the resulting ERC signals were squared, rendering them all positive.

### Resting-state analysis

To examine whether HD-TDCS had any effects on spontaneous neural activity in the stimulated cortex, unrelated to finger movement, we conducted power spectral analysis on data extracted from the rest blocks. We divided the rest periods into arbitrary epochs of 2.5 s, yielding 48 such epochs for each condition (e.g., pre aTDCS, early post aTDCS, etc). In our experience, this epoch length is ideal for resting state EEG/MEG data, giving good frequency resolution while still maintaining enough epochs for reliable estimates of spectral power. Epochs were baseline corrected and bandpass filtered at 0–80 Hz. We computed beamformer weights for voxels on a 10 mm 3-D grid covering the whole brain model, and then projected the full single-trial timecourses of the resting epochs into source space. Spectral density estimates were computed for each epoch using the multitaper method, and then averaged across trials within conditions. This was computationally feasible because it was done on one voxel at a time, and only the results of the power spectral analysis were saved for each voxel, rather than the full single-trial timecourse. Power spectra were then extracted for each motor cortex by averaging within the ROIs.

### Amplitude normalization

After averaging ERCs and power spectra across voxels in the ROIs, there was considerable variation in the amplitude of these signals across subjects. Such variation is not an issue for analyses of ERSP, as that measure is inherently normalized at each frequency as a ratio of power in the active window vs. the baseline, but no such normalization is involved in extraction of ERCs and power spectra. There are many explanations for the variability, including the orientation of the active neurons relative to the sensors, and differences in sensor noise that affect the normalization of the beamformer weights. Without correction for such differences, between-subject averages and statistical comparisons will be dominated by the subjects with largest amplitudes, masking any consistent differences between conditions. We therefore adopted a normalization technique introduced in a prior study of ERCs recorded in MEG (Barca et al., 2011), and used previously by our group for the same purpose (Chu and Meltzer, 2019). For each ROI, the mean and standard deviation across all samples (time points or frequencies) are computed, pooling across all of the conditions that are to be compared. The original data series is then z-scored by subtracting the mean and dividing the result by the standard deviation. This produces appropriately scaled signals in each individual suitable for statistical comparison between conditions that are repeated measures within subjects. The same normalization procedure was applied to ERCs and power spectra prior to the cluster analysis. A separate normalization was performed for each ROI (left and right M1) and hand movement condition (left and right hands), but data were pooled across timepoints (pre-, early post-, late post-stimulation) and stimulation conditions (cTDCS, aTDCS) for each participant.

### Statistical analysis

The primary hypothesis of this experiment was that HD-TDCS would modulate neural activity in opposite directions relative to the pre-stimulation baseline dependent on its polarity, anodal vs. cathodal, and specifically within the early post-TDCS time period. Therefore, primary statistical analyses were based on a planned comparison using a double subtraction:


$$\begin{array}{l} {}[(Earlypost-aTDCS-pre-aTDCS)-\\ \;(Earlypost-cTDCS-pre-cTDCS)]. \end{array}$$

That is to say, we hypothesized that the *difference* of post-aTDCS minus pre-aTDCS (effects induced by the anodal stimulation) would be greater or lesser than the *difference* for post-cTDCS minus pre-cTDCS (effects induced by the cathodal stimulation). This is not a directional hypothesis, as a difference in either direction is compatible with the neural gain or neural economy hypothesis; therefore, two-tailed tests were used. We used this comparison as our primary outcome measure for its parsimony; it avoids having to conduct multiple tests on the effects of anodal and cathodal stimulation separately. On the other hand, a significant difference does not necessarily prove that the changes compared to pre-stimulation were in opposite directions; they could be in the same direction to different degrees. Therefore, we also visually evaluated all significant clusters to ensure that they did indeed arise from changes in opposite directions compared to the pre-stimulation time periods. We only predicted responses to TDCS for movements of the right hand, corresponding to the stimulation location in the left motor cortex; however for completeness we also present the results of analysis of the left hand, corresponding to the unstimulated right motor cortex.

All statistical comparisons were performed on data derived from whole brain SAM maps, averaged across the 150 voxels in each ROI. For analyses of induced changes in spectral power (ERSP), we conducted a paired *t*-test across subjects, focusing on activity within each prespecified time-frequency window. For analyses of ERCs, we were able to analyze each time point separately, using a paired *t*-test at each time point, but this required correction for multiple comparisons across the time points. For this, we used the cluster-based permutation analysis (Maris and Oostenveld, 2007) implemented in FieldTrip software. This non-parametric method essentially constructs a histogram of a test statistic by randomly permuting the data many times (1,000 times is typical) and computing a statistic for each random partition. The test statistic (in this case, Student's paired *t*-test) is then computed for the original non-permuted data and a *p*-value is obtained by comparing this statistic with the permutation distribution. This approach allows one to identify significant clusters (across time in this case) while correcting for multiple comparisons across the time points. We used a timepoint-wise threshold of *p* < 0.025 to detect significant timepoints of positive or negative modulation, yielding a *p* < 0.05 error rate for a two-tailed test. To be accepted as significant, adjacent significant timepoints had to form a large enough cluster of temporal extent to yield a two-tailed family wise error rate of *p* < 0.05. The same method was used for analysis of changes in resting-state power spectra, but clustering across frequencies instead of time points.

## Results

### Behavioral

Overall, TDCS did not seem to affect motor behavior in the task in any specific way, although the task, a simple button-press, was not optimized to detect behavioral effects. We analyzed reaction time based on the measured EMG onsets following the color change cues, using a repeated measures ANOVA (*n* = 18) with within-subject factors of Hand (left, right), stimulation (aTDCS, cTDCS), and timepoint (pre, early post, late post). The only significant effect was a main effect of timepoint [*F*_(2, 34)_ = 4.84, *p* = 0.017, ges = 0.0166]. Visual analysis of average reaction times (Table 1) suggested that response latency decreased in the early post-stimulation period, but returned approximately to baseline in the late post-stimulation period. This impression was confirmed with *post-hoc* pairwise t-tests, corrected with the Holm method, showing that RT was lower in the early post timepoint compared to the pre-stimulation timepoint [*t*_(34)_ = −2.35, *p* = 0.049, mean difference = −43 ms], and compared to the late post-stimulation timepoint [*t*_(34)_ = −2.94, *p* = 0.017, MD = −53 ms], but not significantly different between the pre- and late post-stimulation timepoints [*t*_(34)_ = 0.59, *p* = 0.559, MD = +11 ms]. The lack of interaction effects indicates that this pattern occurred for both the left hand, which corresponded to the unstimulated right motor cortex, and the right hand, corresponding to the stimulated left motor cortex. Furthermore, it occurred regardless of whether stimulation was anodal or cathodal. We believe that the most likely explanation is that participants got faster with practice, but then slowed down again due to fatigue over the course of the experiment.

**Table 1:** Reaction times (ms, mean ± SD) by hand, stimulation condition, and timepoint.

**Hand**	**Stimulation**	**Pre-**	**Early post-**	**Late post-**
Left	Anodal	220 ± 45	213 ± 45	225 ± 59
Right	Anodal	226 ± 36	214 ± 47	229 ± 61
Left	Cathodal	227 ± 39	222 ± 33	233 ± 40
Right	Cathodal	238 ± 48	219 ± 41	234 ± 44

### Event-related currents

**Figure 3:**
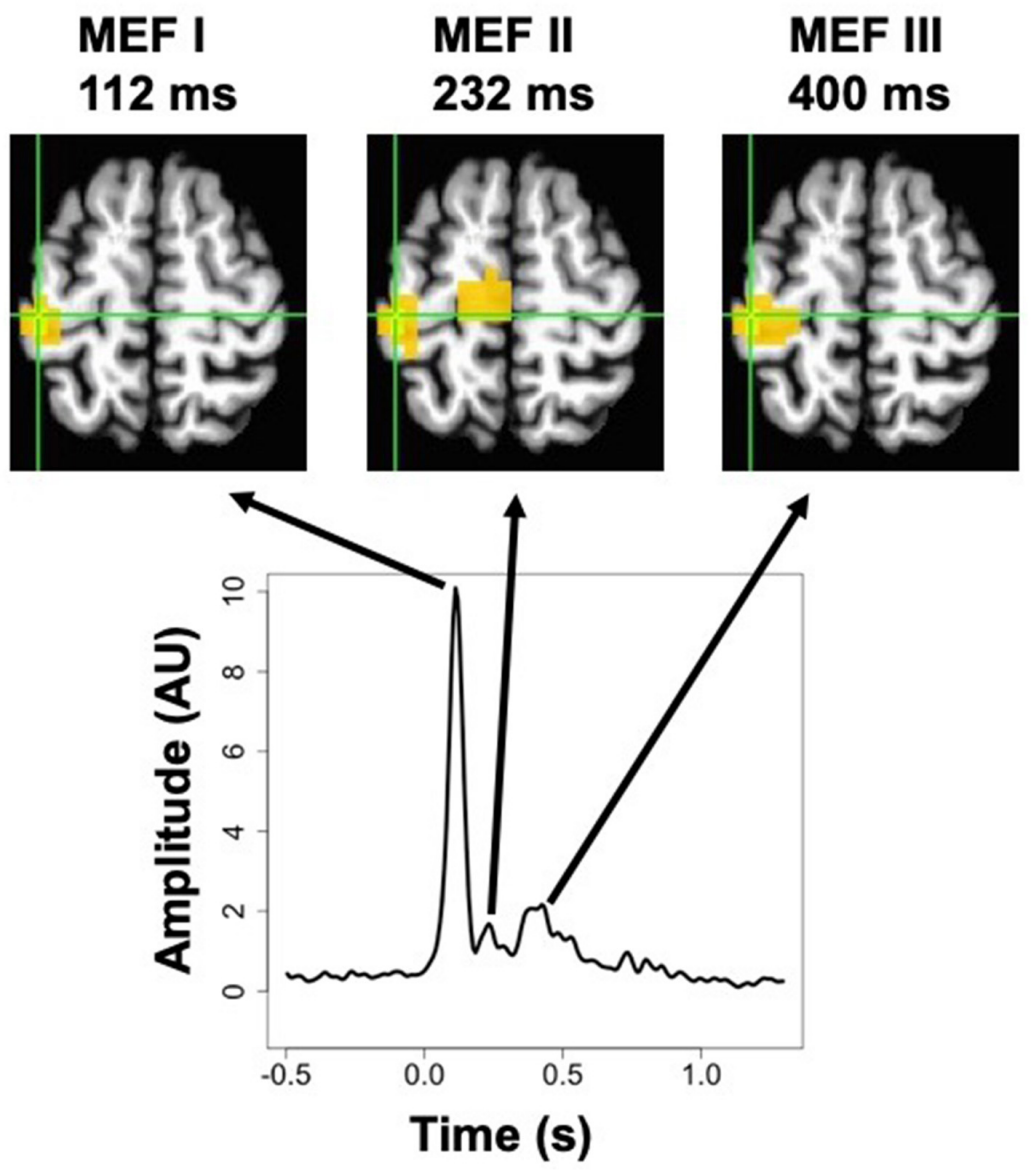
MEG activation maps (thresholded at 50% of the maximum response) for the three peaks in the post-movement event-related current signal, known as Motor Evoked Fields MEFI, MEFII, and MEFIII, for right-hand movements in the pre-stimulation cathodal TDCS condition. Shown below is the averaged timecourse for the peak voxel, which was the same location for all three peaks. The more medial activation for MEFII is located in the paracentral lobule, and forms part of a contiguous cluster with the peak voxel, but the continuity is not visible at this slice (MNI *z* = +60 mm).

The event-related SAM procedure produced whole-brain maps of averaged ERC amplitude at each time point. Inspection of these maps averaged across subjects revealed that three distinct peaks in the signal could be discerned. Although the relative magnitude of each peak varied across voxels, the peak location of all three peaks was exactly the same, at a voxel located near the border of the pre-central and post-central gyri (MNI coordinates *x* = −43, *y* = −26, *z* = +60). The averaged signal at this peak voxel (from the pre-stimulation timepoint in the cathodal TDCS condition) is presented in Figure 3, along with spatial maps thresholded at 50% of the maximum value at each peak, showing a very similar localization for all three. These three peaks occurred at 112, 232, and 400 ms, and correspond very well to the MEFI, MEFII, and MEFIII responses characterized in previous MEG studies of finger movements (Cheyne et al., 2006; Suzuki et al., 2013). We did not find any sign of evoked activity occurring *before* the EMG onset, such as the readiness fields or motor field, but these are more commonly observed with self-paced movements and may not be as readily discernible in cued movements such as those used in this experiment (Suzuki et al., 2013).

Although the peak voxel for the ERC responses was located at the posterior border of motor cortex, all three discernible peaks extended well into the precentral gyrus in their localization, and overlapped extensively with the regions showing beta and gamma ERS, so for the sake of simplicity we elected to conduct statistical analysis on the same 150-voxel ROI for all comparisons of the effects of anodal and cathodal TDCS.

**Figure 4:**
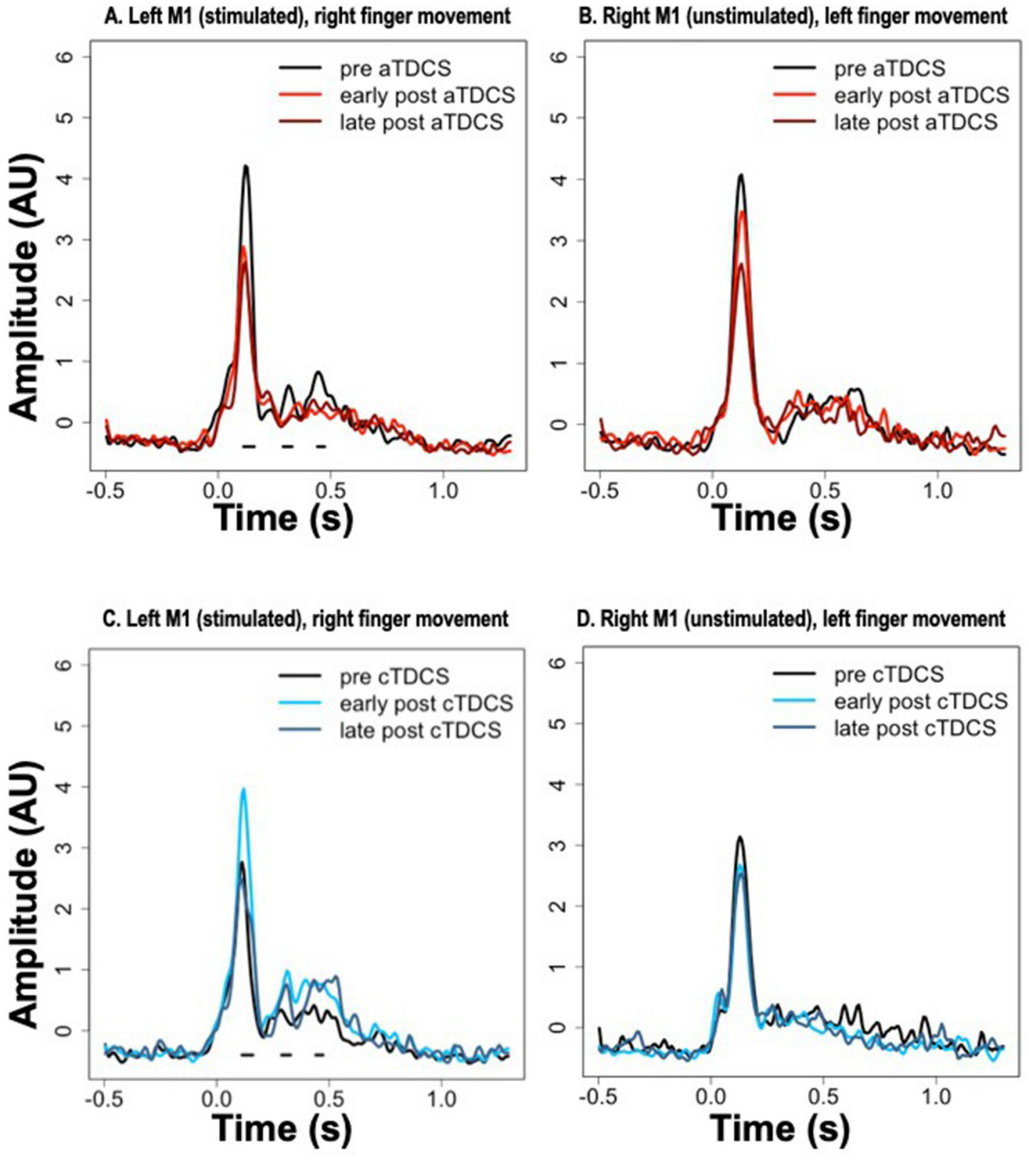
Event-related current timecourses for the three experimental timepoints. Horizontal black lines below the signals show the timepoints at which statistically significant differences occurred in the modulation of the signal after anodal TDCS (aTDCS) (**A**: Left M1, **B**: Right M1) vs. cathodal TDCS (cTDCS) (**C**: Left M1, **D**: Right M1), during the “early post” period compared to the “pre” period. The “late post” period was not included in the statistical analysis. Note that the time periods of significant differences in **(A, C)** are the same, because the statistical comparison is between the data presented in (**A**, anodal) and (**C**, cathodal).

Average timecourses of ERCs for contralateral finger movement are shown in Figure 4 for the stimulated (left) and unstimulated (right) M1 ROIs, at all three time periods—pre, early post, and late post, in both aTDCS and cTDCS sessions. As described in the methods, we focused on a planned statistical comparison testing for differences in the TDCS response between anodal and cathodal stimulation, based on the difference between the pre-stimulation and the early post-stimulation timepoint. This was implemented as a cluster analysis testing for significant consecutive differences across time points. The cluster analysis returned three distinct clusters in the left M1 for right finger movements, which corresponded precisely with the three detected peaks in the ERC response. For all three peaks, aTDCS reduced the magnitude of the ERC response (Figure 4A), while cTDCS increased it (Figure 4C). The first peak, corresponding to the MEF1 response, had a cluster-wise significance of *p* < 0.001, with a temporal extent of 112–160 ms. The second peak, corresponding to the MEFII, was significant at *p* = 0.0059, with a temporal extent of 288–328 ms, and the third, MEFIII, was significant at *p* = 0.024, occurring at 440–472 ms.

Although we did not plan on a direct statistical analysis of the late post-TDCS time period, visual inspection of the plots suggests that the enhancement of the MEFI response by cTDCS is relatively short-lived, as it returns to pre- stimulation levels in the late period (Figure 4C), whereas the enhancement of the MEFII and MEFIII responses may be longer lasting, as these responses remain elevated in the late post- period (Figure 4C). In contrast, the suppression of the MEF responses by aTDCS seems to be consistently long-lasting, as all three peaks are suppressed to similar levels in both the early post and late post periods, relative to the pre- period (Figure 4A).

The same cluster analyses were conducted for the unstimulated right M1 during left finger movements, but did not return any significant clusters. Responses in all three time periods appear very similar in the cTDCS session (Figure 4B). For the aTDCS session (Figure 4D), a slight reduction in the MEFI response is apparent in the early post-stimulation session, becoming more pronounced in the late post-stimulation session, but the early reduction was not significant, and no difference is apparent in the MEFII or MEFIII periods.

### Induced power changes

**Figure 5:**
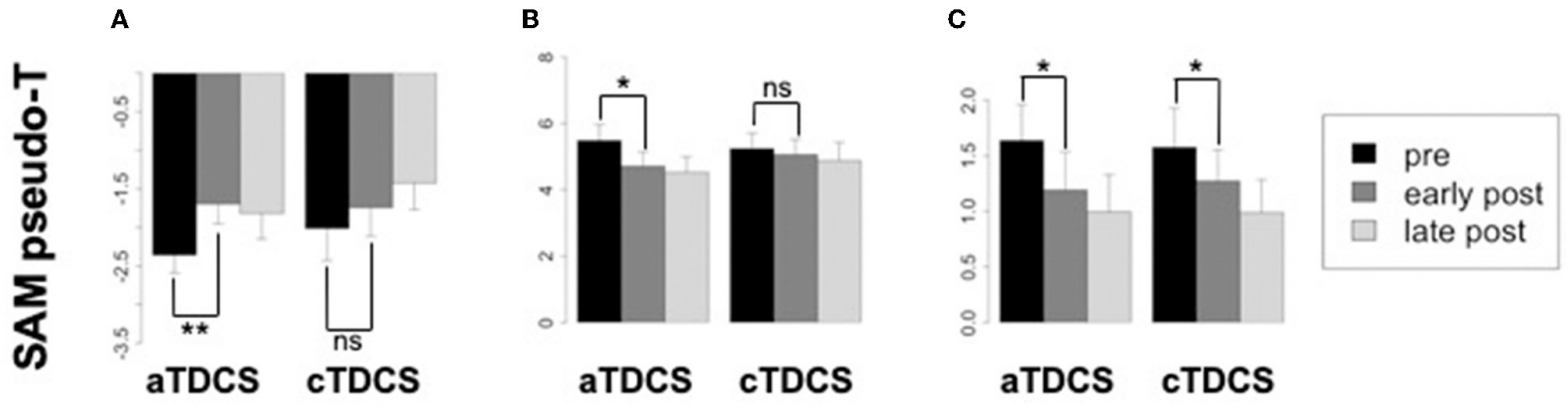
Changes in oscillatory power from the stimulated left primary motor cortex (M1) corresponding to the beta ERD, beta ERS, and gamma ERS time-frequency windows, in each time period relative to aTDCS and cTDCS. Significance codes: **p < 0.01, *p < 0.05, non-significant (ns) *p* > 0.05. Confidence intervals are within-subjects standard errors of the mean computed with the method of Morey (2008) using the R package Rmisc. **(A)** Beta ERD. **(B)** Beta ERS. **(C)** Gamma ERS.

Averaged ERSP responses for beta ERD, beta ERS (rebound), and gamma ERS are shown for the stimulated left M1 ROI during right finger movements, in Figure 5. The planned comparison contrasting the effects of aTDCS and cTDCS did not show any significant polarity-dependent differences in any band. In general, for all bands, responses decreased after stimulation with both aTDCS and cTDCS. For beta ERD (Figure 5A), we observed a significant decrease for early post-aTDCS vs. pre-aTDCS [*t*_(17)_ = 3.88, *p* = 0.001]. Note that the *t*-value is positive because bERD is a negative quantity, so a reduced ERD magnitude results in a more positive number. For cathodal stimulation, we also observed a numerical decrease in magnitude (a more positive number) for early post-cTDCS vs. pre-cTDCS, but it was not significant [*t*_(17)_ = 1.34, *p* = 0.198]. To detect polarity-dependent changes, the most crucial comparison is the direct contrast of these two effects, i.e., the difference-of-differences approach used in our analysis of ERCs, testing whether the early post vs. pre-stimulation changes are different for anodal vs. cathodal stimulation. In this case, they were not significantly different [*t*_(17)_ = 1.38, *p* = 0.18], suggesting that the observed changes were similar under both kinds of stimulation.

Similar results were obtained for the beta rebound ERS (Figure 5B), a positive quantity. A similar reduction for both anodal and cathodal stimulation was observed. Again, the comparison of early post- vs. pre-stimulation ERS was significant for aTDCS [*t*_(17)_ = −2.64, *p* = 0.017] but not for cTDCS [*t*_(17)_ = −0.667, *p* = 0.512]. However, as with the ERD findings, the difference between the changes in the aTDCS vs. cTDCS conditions was not significant [*t*_(17)_ = −1.26, *p* = 0.22], suggesting that these changes were not polarity-dependent.

For gamma ERS (Figure 5C), we observed significant reduction of the ERS response for early post- vs. pre-stimulation timepoints for both aTDCS [*t*_(17)_ = −2.67, *p* = 0.016] and for cTDCS [*t*_(17)_ = −2.66, *p* = 0.016], but these changes were also not significantly different from each other [*t*_(17)_ = −0.644, *p* = 0.528], again indicating a lack of polarity-dependent modulation.

### Effects on resting state power

**Figure 6:**
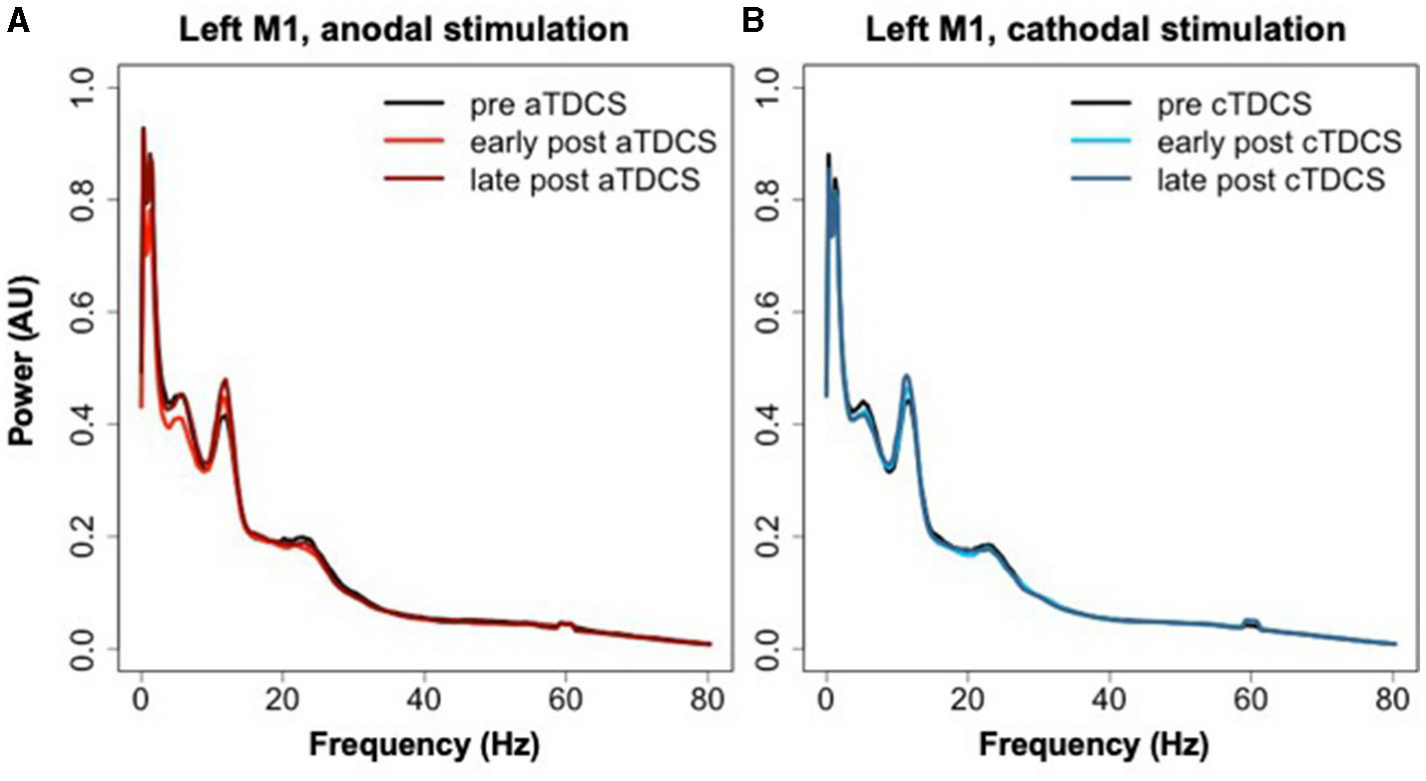
Power spectra extracted from left M1 during rest blocks in the three time periods relative to **(A)** aTDCS and **(B)** cTDCS.

The averaged power spectra for the stimulated left M1 ROI before and after TDCS in both sessions are shown in Figure 6. The cluster analysis returned no significant clusters testing for a contrasting effect of cTDCS vs. aTDCS. For aTDCS, there appeared to be a slight reduction in low-frequency power in the early post- period, but this was not statistically significant (Figure 6A). Power spectra before and after cTDCS are virtually identical (Figure 6B).

## Discussion

This study aimed to distinguish between two possible effects of polarity-dependent TDCS on task-related neural activity associated with finger movements. According to the “neural gain” hypothesis, the increase in motor cortex excitability associated with anodal TDCS should also result in an augmentation of signals associated with voluntary movement, while the inhibition of the same area through cathodal TDCS should have the opposite effect. Under the “neural economy” hypothesis, these predictions are reversed. We conducted planned contrasts testing for polarity-dependent changes, reasoning that either hypothesis could be supported by pre-stimulation vs. post-stimulation changes going in opposite directions after anodal and cathodal TDCS. We examined three kinds of signals: spontaneous oscillations, event-related spectral perturbation, and source-localized time-domain event-related currents (analogous to event-related potentials). Only the event-related currents exhibited polarity-dependent changes, and these supported the neural economy hypothesis.

Specifically, we observed polarity-dependent modulation of the amplitude of event-related currents in three time-windows, corresponding to three successive peaks in the time domain average response following the onset of EMG activity in voluntary visually cued finger movements. These three time windows were each identified by an unbiased algorithm detecting statistically significant differences between the aTDCS and cTDCS conditions, compared to their pre-stimulation timepoints, but they exactly match the three peaks in the signal. This pattern of 3 peaks following finger movements has been identified in numerous previous studies with MEG, and the peaks have been labeled as either movement-related cortical fields (MRCF) or movement evoked fields (MEF), with the three peaks known separately as MEFI, MEFII, and MEFIII.

The exact nature of the neural activity giving rise to the three MEF peaks is uncertain, but experiments indicate that they are most likely due to proprioceptive sensory feedback provided to the motor cortex via peripheral nerves, rather than being a delayed consequence of motor command output. Evidence for this viewpoint includes the facts that the signals are present following both active and passive movements (Onishi et al., 2013), are delayed when the peripheral limb is cooled to increase nerve conduction time (Cheyne et al., 1997), and appear to have different generators than somatosensory evoked fields (SEF) such as those seen in response to cutaneous electrical stimulation (Onishi et al., 2013). As seen in our study, the first of the three peaks, MEFI, is generally the largest, and not all studies detect MEFII and MEFIII. The latency of MEFI is commonly stated to be around 30–40 ms (Suzuki et al., 2013) after the onset of overt movement, or 80–120 ms after the onset of EMG activity (Cheyne et al., 1997), as used in this study for timelocking. The source of MEFI has been frequently localized to the postcentral gyrus, in area 3a or 3b (Cheyne et al., 2006; Murakami et al., 2010), although some studies have localized it to the precentral gyrus (Suzuki et al., 2013). Still other studies have suggested an origin deep in the central sulcus at the boundary of these two regions (Kristeva-Feige et al., 1996; Oishi et al., 2004). In the current study, the observed peak was almost exactly on the border between precentral and postcentral gyrus, but not particularly deep (see Figure 3). In any case, the MEF peaks seem to play a role in sensorimotor integration and the guidance of movement by sensory feedback originating in muscle spindles (Maezawa et al., 2017). The spatial sources of MEFII and MEFIII have been less extensively characterized, although Suzuki et al. (2013) estimated that all three peaks have the same spatial origin. This is consistent with our results, in that all three peaks, localized with beamforming (as opposed to prior studies that largely used single dipole fits) exhibited their maximal amplitude at the same voxel (Figure 3).

Given that all three peaks in our study exhibited similar polarity-dependent modulation by HD-TDCS, our findings support the idea that MEFI, MEFII, and MEFIII share a common neural mechanism reflecting re-afferent input to sensorimotor cortex from peripheral muscles. Thus, they appear to be more an *input-related* phenomenon in the motor cortex, as opposed to the motor-evoked potentials (MEPs) elicited by single-pulse TMS to motor cortex, which represent corticospinal *output* as measured with EMG in peripheral muscles. Interestingly, the direction of modulation observed for cortical MEFs in response to opposing polarities of TDCS is opposite that observed for peripheral MEPs. A very well-established finding is that anodal TDCS increases MEPs, and cathodal TDCS decreases MEPs, leading to these techniques being characterized as excitatory and inhibitory, respectively. In our study, however, the response of MEFs is the opposite—anodal TDCS decreased their magnitude, and cathodal TDCS increased them. If this finding were considered in isolation, one might be tempted to call anodal TDCS the “inhibitory” technique, and cathodal the “excitatory” one. Overall, this pattern of findings shows that modulation of MEPs does not necessarily generalize to equivalent changes in other measures of neural activity obtained from the same cortical region. Our findings therefore fit more with the “neural economy” hypothesis, in that increasing the excitability of motor cortex (as defined by MEPs) seems to decrease the incremental signal observed in response to reafferent input.

Despite the clear dissociation observed in our data on MEFs/MRCFs, our findings cannot be considered definitive in adjudicating between the neural gain and neural economy hypotheses. A clear picture can emerge only by considering the results of many studies in aggregate. Although it is not our intention here to fully review the vast literature on neural responses to TDCS, we note that even limiting ourselves to studies examining time-domain electromagnetic activity (ERPs and ERFs), examples can be found in line with our findings, contrary to our findings, and with null results from TDCS. Supporting the neural gain hypothesis, a fairly large number of studies have now demonstrated increases in the P300 potential after anodal stimulation, corresponding to behavioral improvements in cognitive control, target detection, and related measures (reviewed by Mendes et al., 2022; Liu et al., 2023; Mertens et al., 2023; Zhou et al., 2023). Some studies have also reported decreased N2 potentials after cathodal stimulation in a go/nogo paradigm (Friedrich and Beste, 2018), and increased auditory N2 (Hanenberg et al., 2019) and mismatch negativity (MMN) responses (Impey and Knott, 2015) after anodal stimulation. Similarly, Reinhart et al. (2016) reported increased visual evoked potentials after anodal TDCS to visual cortex. Interestingly, Chen et al. (2023) found that cerebellar stimulation could produce polarity-dependent modulation of somatosensory MMN responses, increasing with anodal and decreasing with cathodal stimulation of the cerebellum. Findings of no change in ERP amplitudes following TDCS have also been reported. Two studies of anodal stimulation applied to M1 found no change in task-related ERPs (Conley et al., 2016; Faehling and Plewnia, 2016), while one study of anodal stimulation found no change in auditory evoked potentials (Kunzelmann et al., 2018). Given the tendency of negative findings to go unreported, it is likely that many other null results have been obtained in previous investigations.

Findings supporting the neural economy hypothesis in the ERP/ERF literature seem to be more sparse, but some examples have been found. Notably, Johari and Berger (2023) found that cathodal stimulation increased the P300 potential in a speech go/no-go task, a finding that they interpreted as indicating compensatory upregulation of certain neuronal responses following decreased cortical excitability induced by the TDCS, similar to our interpretation of our findings, in which cathodal stimulation increased responses and anodal decreased them. Similar findings have also been reported in studies examining steady-state responses to periodic sensory stimulation, which is also an effect seen in time-domain averages of electromagnetic signals, albeit driven by repetition of stimuli at a specific frequency. Heinrichs-Graham et al. (2017) reported reduced steady-state responses to visual flicker stimulation after anodal stimulation to visual cortex. Pellegrino et al. (2019) reported reduced gamma synchrony in response to 40 Hz auditory stimulation following bilateral stimulation. However, the generators of that activity are rather distant from the stimulation sites, suggesting that the electrophysiological effects of TDCS may ultimately be rather remote from the stimulated areas, and driven by complex polysynaptic mechanisms. In that respect, the present study offers some clarity, as our approach benefited from high spatial resolution in both the stimulation site (concentrated on M1 using HD-TDCS) and the measurement of the neural responses, localized to sensorimotor cortex with beamforming analyses of MEG data, in close agreement with prior studies of the MRCF responses. Thus, we can be fairly confident that, in our study, anodal and cathodal stimulation was accurately applied to the same area that generated the responses that were differentially affected by the two polarities. In contrast, many studies using conventional TDCS involve current flowing through fairly wide areas of the brain to travel between the two electrodes, even stimulating areas that are close to neither of the electrodes but lie in between them (de Berker et al., 2013; Alam et al., 2016). Similarly, the generators of large diffuse ERP responses such as MMN and P300 are not well characterized, whereas the MRCF responses are quite specific to sensorimotor cortex, and are spatially localized with enough precision that they have only been characterized so far in MEG, with no obvious EEG correlate.

In contrast to our clear findings regarding reafferent sensory inputs, we did not observe polarity-dependent modulation in the components of the neural signal that are more associated with motor output, namely beta ERD (thought to be excitatory), beta rebound ERS (thought to be inhibitory, related to cessation of movement), and gamma ERS (excitatory). Rather, all three of these signals tended to decrease after TDCS regardless of the polarity (Figure 5). Unfortunately, it is not possible to determine from our data whether this is a genuine effect related to TDCS in a polarity-independent manner, or rather simply an attenuation of activity related to practice and fatigue as the experiment proceeds. To determine this, it would have been necessary to include a third session using sham TDCS as an additional control. We decided not to do this when we originally designed the experiment, as we believed that three experimental sessions of MEG-TDCS-MEG would be too burdensome for participants, and we would suffer an unacceptably high rate of attrition with people failing to complete all three sessions (four, with the MRI). Thus, the potential of a polarity-independent attenuation of neural activity related to voluntary movement remains to be further investigated in a future study. In any case, this does not invalidate our finding of polarity-dependent modulation of the MEF responses, as contrasting effects of the two polarities were our main target in this study.

The third outcome measure explored in this study was modulation of resting state oscillatory activity, measured during brief resting periods interspersed throughout the task. We did not find any significant differences between aTDCS and cTDCS in resting state power spectra extracted from the motor cortex ROIs. This lack of an effect is not surprising, as a previous study also failed to find any polarity-dependent modulation of resting state oscillations with anodal and cathodal stimulation (Pellicciari et al., 2013). On the other hand, studies with positive results have been quite mixed in the details. For example, Boonstra et al. (2016) found a “slowing” of spontaneous oscillations (shift to lower frequencies) in healthy volunteers, while Marceglia et al. (2016) found instead a “speeding” in patients with Alzheimer's disease, with both studies comparing anodal vs. sham stimulation. Mangia et al. (2014) found an increase in power across low and high frequencies with anodal stimulation, further confusing the picture. Thus, there is no consensus about the effects of TDCS polarity on spontaneous EEG oscillations; future studies and systematic reviews should attempt to determine the role played by various factors such as electrode location, current distribution, intensity, and duration.

Several limitations may be noted about this study in addition to those mentioned above. First, our study was limited to young adults and was therefore unable to reveal any potential effects of aging. Additionally, the study was not sufficiently powered to reveal sex differences and had an imbalanced ratio, with twice as many female participants as male. Another methodological consideration is participant blinding, and the assessment of whether or not participants are aware of the differences between stimulation conditions. We did not formally assess this. However, this is mainly a concern for studies comparing active stimulation to sham, as many participants can distinguish these, especially if they are not naive to TDCS (Ambrus et al., 2012), or if stimulation is at higher intensities (O'Connell et al., 2012). In contrast, participants are not generally able to distinguish anodal vs. cathodal stimulation (Tang et al., 2016), and this is consistent with our own anecdotal experience with the techniques—to us, they feel exactly the same. In the present study, we only compared anodal vs. cathodal stimulation (see above discussion on the lack of a sham condition), so we do not believe that blinding is a major concern.

In summary, we found that anodal and cathodal HD-TDCS produced opposite modulation of movement-related cortical fields, in line with the neural economy hypothesis. That is, anodal stimulation, thought to increase cortical excitability, decreased the amplitude of reafferent responses in the motor cortex, while cathodal stimulation, thought to be inhibitory, increased them. The high spatial precision of HD-TDCS combined with MEG allows us to be fairly confident that these effects are specific to the stimulated area, rather than being a result of a complex polysynaptic mechanism spanning multiple brain regions. More investigation is needed to clarify the effect of noninvasive brain stimulation on neuronal activity local and remote to the stimulation site. We believe that the approach employed in this study can be fruitfully applied to investigating a wider variety of neural responses originating from different brain regions beyond the motor cortex.

## Data availability statement

The raw data supporting the conclusions of this article will be made available by the authors, without undue reservation.

## Ethics statement

The studies involving humans were approved by Baycrest Hospital Research Ethics Board. The studies were conducted in accordance with the local legislation and institutional requirements. The participants provided their written informed consent to participate in this study.

## Author contributions

JM: Conceptualization, Funding acquisition, Investigation, Methodology, Software, Supervision, Visualization, Writing – original draft, Writing – review & editing. GS: Investigation, Writing – review & editing. TD: Investigation, Project administration, Supervision, Writing – review & editing. MZ: Investigation, Writing – review & editing. CL: Investigation, Writing – review & editing. FF: Conceptualization, Funding acquisition, Writing – review & editing. AF-N: Conceptualization, Formal analysis, Methodology, Writing – original draft, Writing – review & editing.
